# Dissection of pyroptosis-related prognostic signature and CASP6-mediated regulation in pancreatic adenocarcinoma: new sights to clinical decision-making

**DOI:** 10.1007/s10495-023-01823-7

**Published:** 2023-03-07

**Authors:** Jun Zhu, Yanlong Shi, Sheng Lan, Jingyan Wang, Fei Jiang, Caiping Tang, Yuan Cai, Ziyuan Pan, Haokun Jian, Hanlu Fang, Yewei Zhang, Fei Zhong

**Affiliations:** 1 Department of Oncology, Fuyang Hospital of Anhui Medical University, Fuyang, 236000 Anhui China; 2 Hepatopancreatobiliary Center, The Second Affiliated Hospital of Nanjing Medical University, 121 Jiangjiayuan, Nanjing, 210003 Jiangsu China; 3 The Second Clinical College of Guangzhou Medical University, Guangzhou, 510030 Guangdong China; 4 Department of Anesthesia, Shaoxing People’s Hospital, Shaoxing, 312000 Zhejiang China; 5 Department of General Surgery, Fuyang Hospital of Anhui Medical University, Fuyang, 236000 Anhui China; 6 The First Clinical Medicine College of Guangdong, Medical University, Zhanjiang, 524000 Guangdong China; 7 School of Public Health, Guangzhou Medical University, Guangzhou, 511400 Guangdong China; 8 Hunan University of Chinese Medicine, Changsha, 410000 Hunan China; 9 School of Basic Medical Sciences, Xinxiang Medical University, Xinxiang, 453003 Henan China; 10 Institute of Medical and Health Science, Hebei Medical University, Shijiazhuang, 050017 Hebei China; 11 Department of Oncology, The First Affiliated Hospital of Anhui Medical University, No.81, Meishan Road, Hefei, 230000 Anhui China

**Keywords:** Pyroptosis, Caspase 6, Pancreatic adenocarcinoma, Prognosis, Nomogram, Competing endogenous RNA

## Abstract

Recent studies have indicated that pyroptosis may participate in the regulation of tumorigenesis and immune microenvironment. However, the role of pyroptosis-related genes (PRGs) in pancreatic adenocarcinoma (PAAD) remains unclear. Through multiple bioinformatics analysis, we constructed a prognostic gene model and competing endogenous RNA network. The correlation between PRGs and prognosis, immune infiltration, immune checkpoints, and tumor mutational burden was analyzed by Kaplan–Meier curve, univariate Cox, multivariate regression, and Spearman’s analysis in PAAD patients. The qRT-PCR, Western blotting, CCK-8, Wound healing, and Transwell assay were applied to examine the role of CASP6 in PANC-1 cell. Thirty-one PRGs were upregulated in PAAD. Functional enrichment analysis revealed that the PRGs were mainly involved in pyroptosis, NOD-like receptor signaling pathway, and response to bacteria. We established a novel 4-gene signature related to PRGs for evaluating the prognosis of PAAD patients. Patients with PAAD in the low-risk group had a better prognosis than those in the high-risk group. The nomogram suggested that the 1-, 3-, and 5-years survival probability exhibited robust predictive performance. Significant correlation was observed between prognostic PRGs and immune infiltration, immune checkpoints, and tumor mutational burden. We first identified the potential competing endogenous RNA regulatory axis in PAAD: lncRNA PVT1/hsa-miR-16-5p/CASP6/CASP8. Moreover, knockdown of CASP6 dramatically inhibited the proliferation, migration, and invasion ability of PANC-1 cell in vitro. In conclusion, CASP6 could be a potential biomarker, promoting the occurrence and progression in PAAD. The lncRNA PVT1/hsa-miR-16-5p/CASP6/CASP8 regulatory axis plays an vital role in regulating the anti-tumor immune responses for PAAD.

**Supplementary Information:**

The online version contains supplementary material available at 10.1007/s10495-023-01823-7.

## Introduction

Pancreatic adenocarcinoma (PAAD) is one of the most malignant digestive tumors and is characterized by a low resection rate, high metastasis and recurrence rate, and is especially insensitive to radiotherapy and chemotherapy [1–3]. Its incidence is increasing in young women aged 15 to 34 years, becoming the second leading cause of cancer related death by 2030 [4, 5]. Most patients with PAAD have atypical symptoms before reaching an advanced stage, which greatly reduces the success rate of diagnosis and treatment [6]. The 5-year overall survival (OS) rate is approximately 5% [7]. Although many predictive biomarkers related to PAAD have been studied, they cannot fully meet the requirements for individualized treatment and survival prediction. Therefore, new prognostic models and predictive markers are required to enhance the survival rate of patients with PAAD.

As a form of programmed cell death that depends on inflammasomes, pyroptosis is characterized by the formation of holes in the plasma membrane, resulting in the swelling and rupture of cells and the escape of pro-inflammatory contents [8, 9]. This form of cell death mainly occurs in specialized phagocytes, such as macrophages, monocytes, and DCs, but emerging evidence suggests that it can also be induced in other cell types [10]. A growing number of studies have revealed that pyroptosis could be a double-edged effect of promotion and inhibition of chronic diseases and tumors [11, 12]. The effectors of pyroptosis, cleavage of GSDMD and GSDME, lead to pore formation and secretion of IL-1β and IL-18 from the cytoplasm into the microenvironment [13]. Moreover, the presence of IL-1β and IL-18 can provide conducive conditions for tumor progression [14]. Recently, the pyroptosis-related gene (PRG) signature has played a vital role in various cancers and the potential mechanisms involve noncoding RNAs [15, 16]. However, there are no reports on the relationship between PRGs and competing endogenous RNA (ceRNA) networks in PAAD.

In this study, we screened PRG expression and prognosis to construct a novel signature for PAAD, followed by nomogram creation and calibration to assess accurate values. Moreover, we investigated the molecular functions and relevant regulatory axis of PAAD. These results reveal novel prognostic biomarkers for patients with PAAD and provide a potential therapeutic basis.

## Material and Methods

### Data acquisition

The RNA sequencing data of 486 PAAD patients and the corresponding clinical information were obtained from TCGA database on April 1, 2021. The clinical information of the PAAD patients is shown in Table 1. Data processing was performed using the R software (version 4.0.3). A total of 33 PRGs were obtained from Genecards database (https://www.genecards.org/) (Supplementary Table 1).

**Table 1:** The clinical information of pancreatic adenocarcinoma patients in TCGA

Clinical characters	Number
Gender	
Male	99
Female	80
Status	
Alive	86
Dead	93
Age	
Mean (SD)	64.6 (10.9)
Median [Min, Max]	65 [35,88]
pTNM	
I	21
II	147
III	3
IV	5
T stage	
T	
T1	7
T2	24
T3	143
T4	3
TX	1
N stage	
N	
N0	50
N1	124
Nx	4
M stage	
M	
M0	80
M1	5
Mx	94
Grade	
G1	31
G2	96
G3	48
G4	2
GX	2

### Identification of differentially expressed PRGs

The R “limma” and “ggplot2” packages were applied to explore differential expression between PAAD and normal tissues. A protein–protein interaction network (PPI) of PRGs was constructed using STRING database (https://string-db.org/). The correlation among 33 PRGs was conducted by R “pheatmap” packages.

### Analyses of functional enrichment

Gene ontology (GO) and Kyoto Encyclopedia of Genes and Genomes (KEGG) enrichment analyses were performed using the Metascape database (http://metascape.org/gp/index.html#/main/step1).

### Establishment and development of a PRG-related prognostic gene signature

The correlation between PRG expression and OS in PAAD patients was evaluated using Cox regression analysis. We selected PRGs with significant prognostic value for further analysis and used LASSO Cox regression analysis to build a prognostic model. According to the median risk score, PAAD patients were divided into low- and high-risk groups, and the risk score was calculated as follows: Risk score = 0.300 × CASP4 expression + 0.083*GSDMC expression + 0.079*IL18 expression + 0.052*NLRP2 expression. The Kaplan–Meier analysis was conducted to compare the OS between the low- and high-risk groups. Univariate and multivariate Cox regression analyses were applied to validate the effectiveness of the risk score and threshold value as hazard ratios (HR) and 95% confidence intervals (95% CI). The predictive accuracy of prognosis indicators was evaluated by plotting a time-dependent receiver operating characteristic (ROC) curve. The P-values, HRs, and 95% CIs for each variable were displayed using the "forestplot" R package. For further development, we built a nomogram to predict the 1-, 2-, and 3-year overall survival rates. Moreover, a calibration curve was used to verify consistency.

### Analysis of TMB, immune checkpoints, and immune infiltration

Spearman’s correlation analysis was used to calculate the correlations between prognostic PRG expression and TMB score and two immune checkpoint pathway genes (inhibitory and stimulatory) in PAAD, with the results presented in bubble charts. To further determine the relationship between PRG expression and immune activity, immune infiltration was analyzed using the “Gene” module of the TIMER2 database.

### Identification of CeRNA regulatory axis

To further investigate the underlying functions, we constructed a ceRNA network to elucidate the molecular mechanisms involved. The miRNA targets binding to PRGs were identified using TarBase V.8 (https://dianalab.e-ce.uth.gr/html/diana/web/index.php?r=tarbasev8). We then identified the upstream lncRNAs of miRNAs using miRNet (https://dianalab.e-ce.uth.gr/html/diana/web/index.php?r=tarbasev8). The expression and prognosis of miRNAs and lncRNAs were analyzed using starBase (https://starbase.sysu.edu.cn/index.php) and GEPIA2 (http://gepia2.cancer-pku.cn/#index), respectively.

### Cell culture

Cell lines of PANC-1 was purchased from Procell Life Science (Wuhan, China) and cultured in the Dulbecco’s Modified Eagle Medium (DMEM; C11965500BT; Gibco) culture with 10% fetal bovine serum (FBS; cat no.2127186; VivaCell) and 1% penicillin and streptomycin (cat no.15140122; Gibco). The cell was maintained at 37℃ in a humidified 5% CO2 environment.

### RNAi assay

The siRNA sequences targeting CASP6 used in this study were as follows: siCASP6-1: (sense: 5′-GCAGAUAGAGACAAUCUUAdTdT-3′ and anti-sense: 5′-UAAGAUUGUCUCUAUCUGCdTdT-3′); siCASP6-2: (sense: 5′-CAGAGAAGUUGGACACCAAdTdT-3′ and anti-sense: 5′-UUGGUGUCCAACUUCUCUGdTdT-3′); siCASP6-3: (sense: 5′-GCUUGUUCAAAGGAGACAAdTdT-3′ and anti-sense: 5′-UUGUCUCCUUUGAACAAGCdTdT-3′). siCASP6-1, siCASP6-2, siCASP6-3 were purchased from Hanbio Biotechnology Co., Ltd (Shanghai, China). Transfection was performed using Lipofectamine 3000 (cat no. L3000001; Invitrogen) according to the manufacturers’ instructions.

### Quantitative real-time PCR (qRT-PCR)

Total RNA was extracted using TRIzol reagent (cat no.12183–555; Invitrogen). The cDNA was synthesized with the PrimeScript ™ RT Master Mix kit (cat no.RR036A; Takara). QRT-PCR was implemented utilizing TB Green® Premix Ex Taq™ II (cat no.RR820A; Takara). The primers used were as follows: CASP6 forward: ATGGCGAAGGCAATCACATTT and CASP6 reverse: GTGCTGGTTTCCCCGACAT. Actin forward: 5′-GTGGCCGAGGACTTTGATTG-3′ and Actin reverse: 5′-CCTGTAACAACGCATCTCATATT-3′. The relative expression of CASP6 was determined using the 2 ^− ΔΔCt^ method.

### Western blotting

Cells were lysed with RIPA buffer and proteins were extracted, then protein was quantified with the BCA kit (cat no. P0010; Beyotime). Protein was separated using 10% SDS-PAGE, which was then electrotransferred to PVDF membrane. Next, the PVDF membrane was blocked with 5% bovine serum albumin (BSA) for 1 h at room temperature. The PVDF membranes were incubated with primary antibodies against CASP6 (cat no. ab108335; Abcam) and beta-Actin (cat no. ab8226; Abcam) overnight at 4 ℃, and then incubated with a secondary antibody (Cell Signaling Technology) for 1 h at room temperature. Finally, the ECL solution was added for exposure using chemiluminescence imager.

### CCK-8 assay

Cell counting kit-8 test (CCK-8) was performed to assess the proliferation capabilities of PANC-1 cells. Cells were seeded into a 96-well plate with 3,000 cells per well. 10 ml of CCK-8 reagent was added to the test well and incubated for 2 h at 37 °C. At 6, 24, 48, 72, and 96 h, cell viability was measured by scanning with a microplate reader (Tecan, Switzerland) at a wavelength of 450 nm.

### Wound healing

Cells (3 × 10^5^ cell/pore) were seeded into 6-plate wells for 24 h. Then, the cell monolayers was scratched with the tip of a sterile pipette and the cells were cultured in serum-free medium. Each wound was examined with microscopy (Olympus, Japan) at 0 and 24 h.

### Transwell assay

Cell invasion was assessed by conducting the Chamber matrigel (cat no. 356234; BD Biosciences) invasion 24-well cell according to the manufacturer’s instructions. In the upper chamber, PANC-1 cells (1 × 10^5^/well) were seed into 100 mL serum-free medium, While the lower chamber was filled with 600 µL medium containing 10% FBS. After incubation for 24 h, the invaded cells were fixed in 4% paraformaldehyde, and the 0.1% crystal violet (cat no. C0775; Sigma) was applied for cell staining. Randomly select the field of view on an inverted light microscope (×100 magnification) and count using ImageJ software.

### Statistical analysis

The Wilcoxon rank-sum test was used to analyze the expression of 33 PRGs in PAAD. The data with abnormal distribution were analyzed using Spearman analysis. The relationship between low- and high-risk groups on OS of PAAD patients was analyzed using Kaplan–Meier curves with a two-sided log-rank test. Statistical analyses were performed using R, version 4.0.3. *P* < 0.05 was considered statistically significant.

## Results

### Identification of the expression of PRGs in PAAD

**Fig. 1:**
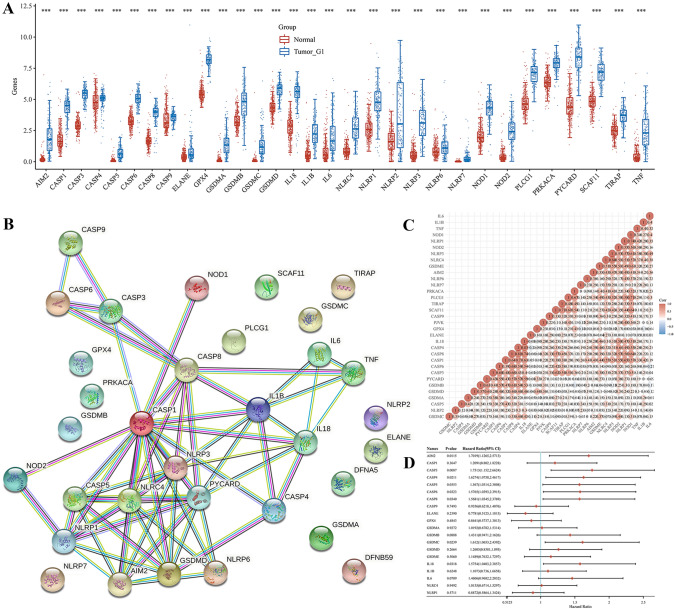
Identification of the expression of PRGs in PAAD. **A** The expression of 33 PRGs in PAAD and normal tissues. Tumour, blue. Normal, red. **B** The PPI network among 33 PRGs. **C** The correlation among 33 PRGs. **D** The forest map presented the prognosis of PRGs in PAAD. ***P < 0.001

We identified the expression of 33 PRGs in PAAD and normal tissues using TCGA and GTEx datasets, resulting in 31 genes being upregulated, while the other two genes (GSDME and PJVK) were not detected (Fig. 1A). Based on the STRING database, the PPI network indicated that AIM2, PYCARD, CASP1, CASP5, CASP8, GSDMD, NLRC4, NLRP1, and NLRP3 were the hub genes (Fig. 1B). Correlation analysis was performed to determine the interactive relationships among these genes (Fig. 1C).

### Functional enrichment analysis of PRGs

To elucidate the role of PRGs, GO and KEGG analyses were performed using Metascape (Table 2). GO molecular function analysis revealed that 33 PRGs were mainly associated with cysteine-type endopeptidase activity involved in the apoptotic process, protein domain specific binding, phosphatidylinositol-4,5-bisphosphate binding, cytokine receptor binding, and caspase activation and recruitment domain binding. GO biological processes analysis suggested that 33 PRGs were mainly involved in pyroptosis, response to bacteria, positive regulation of interleukin-1 beta production, response to lipopolysaccharide, and apoptotic signaling pathways. We also found that these 33 PRGs were mainly involved in the inflammasome complex, cell body, phagocytic vesicle, and secretory granule lumen in GO cellular components analysis. KEGG pathway analysis revealed that 33 PRGs correlated with the NOD-like receptor signaling pathway, Salmonella infection, tuberculosis, apoptosis, and neutrophil extracellular trap formation.

**Table 2:** Significantly GO and KEGG analysis with Metascape

Term	Category	Description	Count
GO: 0019904	GO molecular functions	Protein domain specific binding	11
GO: 0097153	GO molecular functions	Cysteine-type endopeptidase activity involved in apoptotic process	8
GO: 0005126	GO molecular functions	Cytokine receptor binding	8
GO: 0005546	GO molecular functions	Phosphatidylinositol-4,5-bisphosphate binding	6
GO: 0050700	GO molecular functions	CARD domain binding	4
GO: 0009617	GO biological processes	Response to bacterium	23
GO: 0070269	GO biological processes	Pyroptosis	16
GO: 0032496	GO biological processes	Response to lipopolysaccharide	14
GO: 0032731	GO biological processes	Positive regulation of interleukin-1 beta production	13
GO: 0097190	GO biological processes	Apoptotic signaling pathway	10
GO: 0061702	GO cellular components	Inflammasome complex	10
GO: 0044297	GO cellular components	Cell body	5
GO: 0045335	GO cellular components	Phagocytic vesicle	3
GO: 0034774	GO cellular components	Secretory granule lumen	3
hsa04621	KEGG pathway	NOD-like receptor signaling pathway	18
hsa05132	KEGG pathway	Salmonella infection	15
hsa05152	KEGG pathway	Tuberculosis	9
hsa04210	KEGG pathway	Apoptosis	5
hsa04613	KEGG pathway	Neutrophil extracellular trap formation	5

### Construction and evaluation of a PRGs prognostic gene signature

**Fig. 2:**
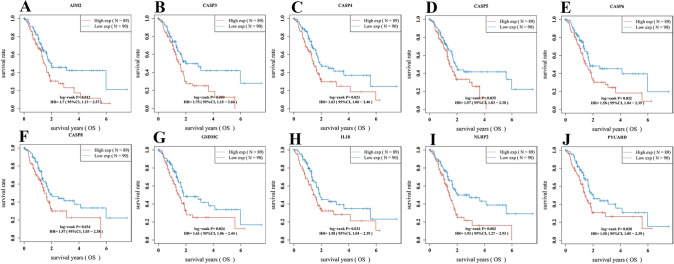
The prognostic value of PRGs between low- and high-expression groups in patients with PAAD. **A** AIM2 **B** CASP3 **C** CASP4 **D** CASP5 **E** CASP6 **F** CASP8 **G** GSDMC **H** IL18 **I** NLRP2 **J** PYCARD

**Fig. 3:**
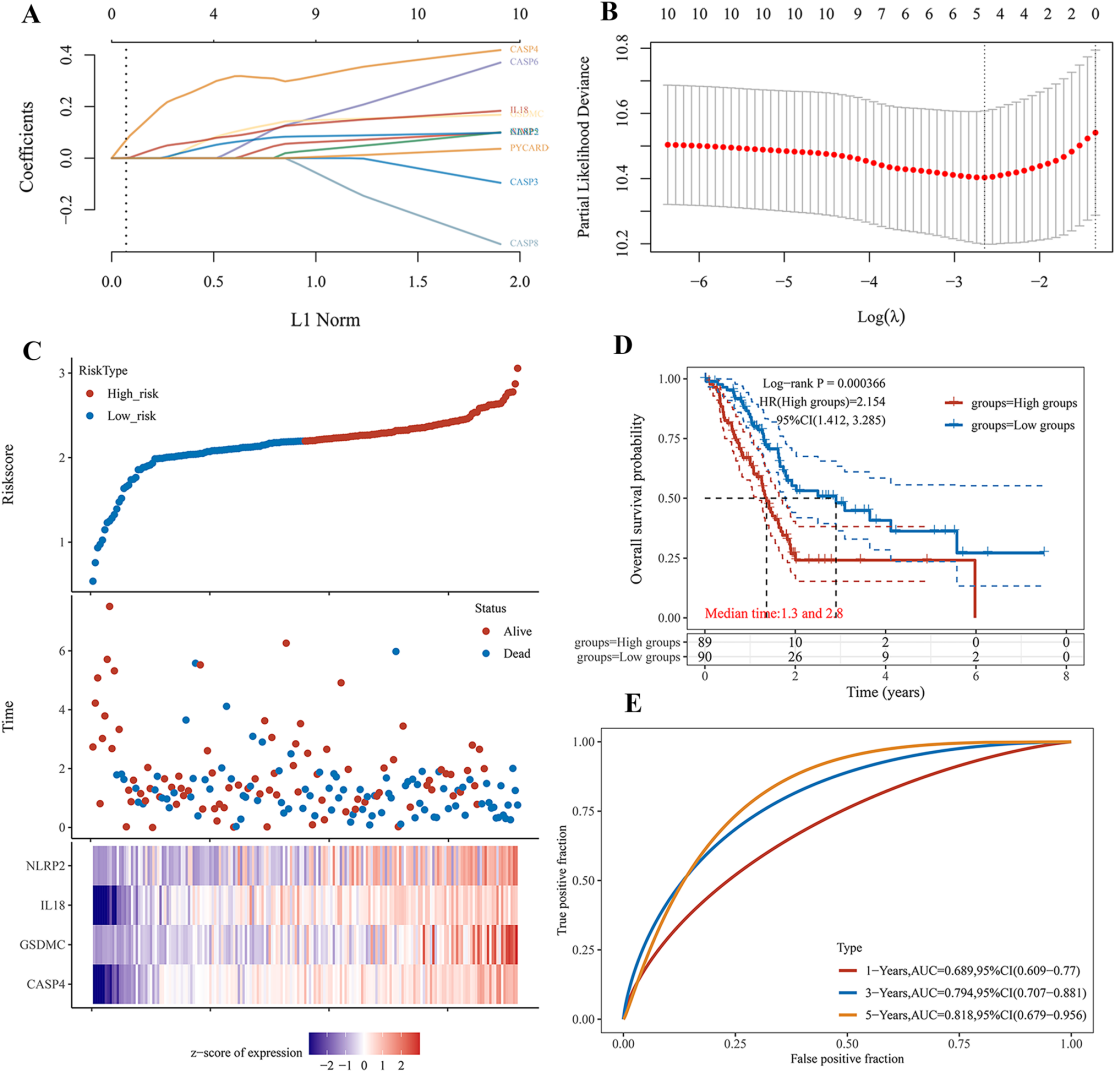
Construction of a novel prognostic PRGs gene signature in PAAD. **A** LASSO coefficient profiles of the 10 PRGs. **B** Plots of the ten-fold cross-validation error rates. **C** Distribution of risk score, survival status, and the expression of 4 prognostic PRGs in PAAD. **D** The overall survival probability between low- and high-expression groups. **E** The AUC time-dependent ROC curves in 1-, 3-, and 5-year

Univariate Cox regression analysis was used to evaluate the prognosis of PRGs in patients with PAAD. We identified 10 genes with clinical prognostic value: AIM2, CASP3, CASP4, CASP5, CASP6, CASP8, GSDMC, IL18, NLRP2, and PYCARD (Fig. 1D). Moreover, Kaplan–Meier curves suggested a longer survival time in PAAD patients with low expression of AIM2 (Fig. 2A, *P* = 0.012), CASP3 (Fig. 2B, *P* = 0.009), CASP4 (Fig. 2C, *P* = 0.021), CASP5 (Fig. 2D, *P* = 0.035), CASP6 (Fig. 2E, *P* = 0.032), CASP8 (Fig. 2F, *P* = 0.034), GSDMC (Fig. 2G, *P* = 0.024), IL18 (Fig. 2H, *P* = 0.032), NLRP2 (Fig. 2I, *P* = 0.002), and PYCARD (Fig. 2J, *P* = 0.028). Based on the prognostic genes mentioned above, we established a novel PRG signature using LASSO Cox regression (Fig. 3A, B). Patients with PAAD were classified into low- and high-risk groups based on their median risk score. We found that a higher risk score was associated with an increased risk of death (Fig. 3C). Next, the Kaplan–Meier curve suggested that patients with PAAD in the low-risk group had a better prognosis than those in the high-risk group (Fig. 3D, *P* < 0.001, HR 2.154, 95%CI 1.412–3.285). Moreover, we further investigated the survival probability using ROC curves, indicating that the area under the curves were 0.689 (95%CI 0.609–0.770) at 1 year, 0.794 (95%CI 0.707–0.881) at 3 years, and 0.814 (95%CI 0.679–0.956) at 5 years (Fig. 3E).

### Development and calibration of a predictive nomogram

**Fig. 4:**
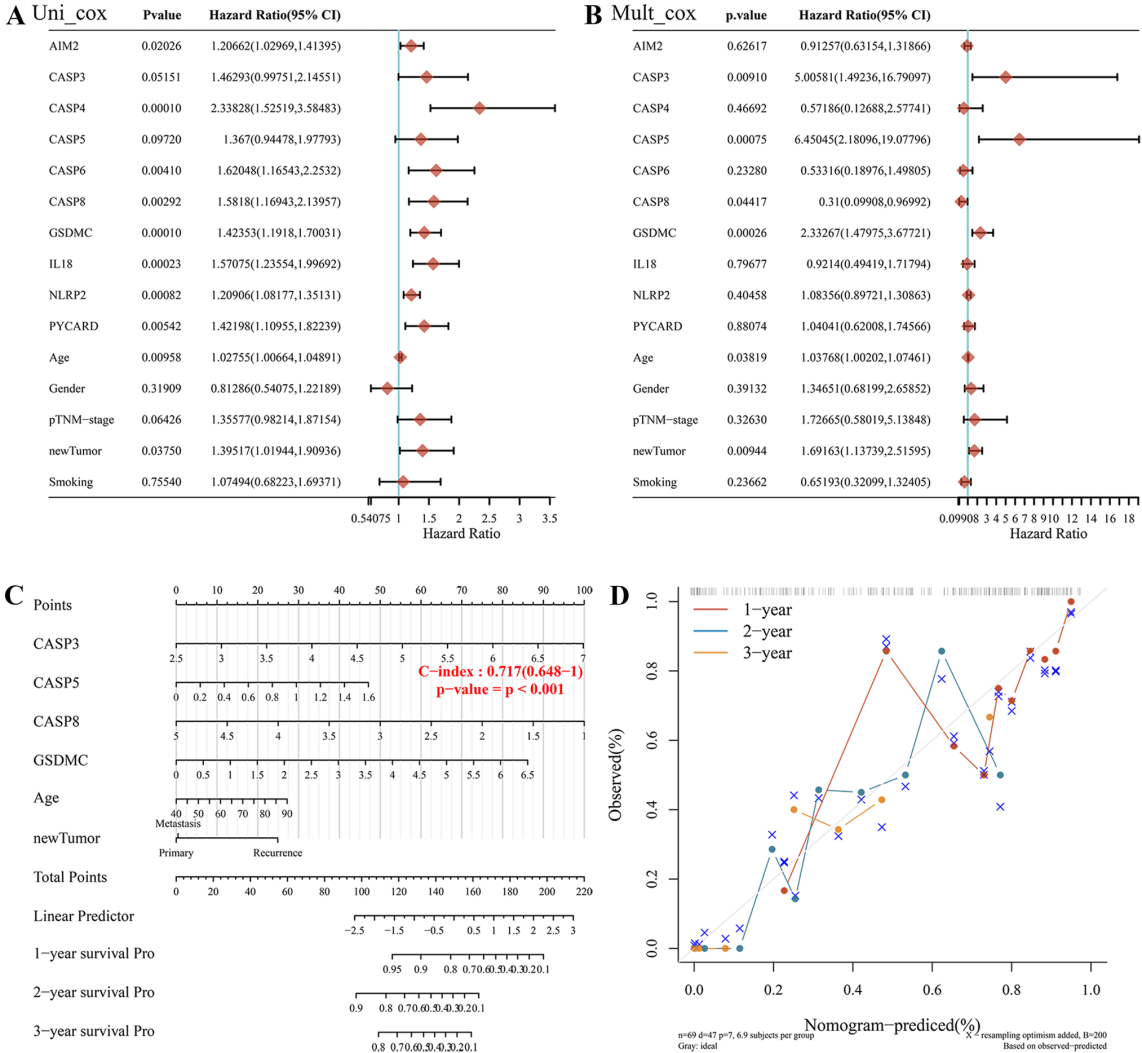
Development of a predictive nomogram. **A** The prognostic value of PRGs and clinical features was analyzed by univariate analysis. **B** The prognostic value of PRGs and clinical features was analyzed by multivariate analysis. **C** Development of a Nomogram to predict the 1-year, 2-year, and 3-year survival probability based on PRGs and clinical features. **D** The calibration curve was to verify the efficacy of nomogram

Combined with prognostic PRGs and clinicopathological characteristics, we implemented a predictive nomogram to predict the probability of survival. Univariate and multivariate regression analyses showed that CASP8, GSDMC, age, and new tumor type were independent factors influencing the prognosis of patients with PAAD (Fig. 4A, B). We then constructed a nomogram for clinical prediction based on CASP3, CASP5, CASP8, GSDMC, age, and tumor status. The nomogram suggested that the 1-, 3-, and 5-years survival probability exhibited robust predictive performance. In addition, the calibration curves showed good consistency with the nomogram (Fig. 4C, D).

### Analysis of TMB, immune checkpoints, and immune infiltration

**Fig. 5:**
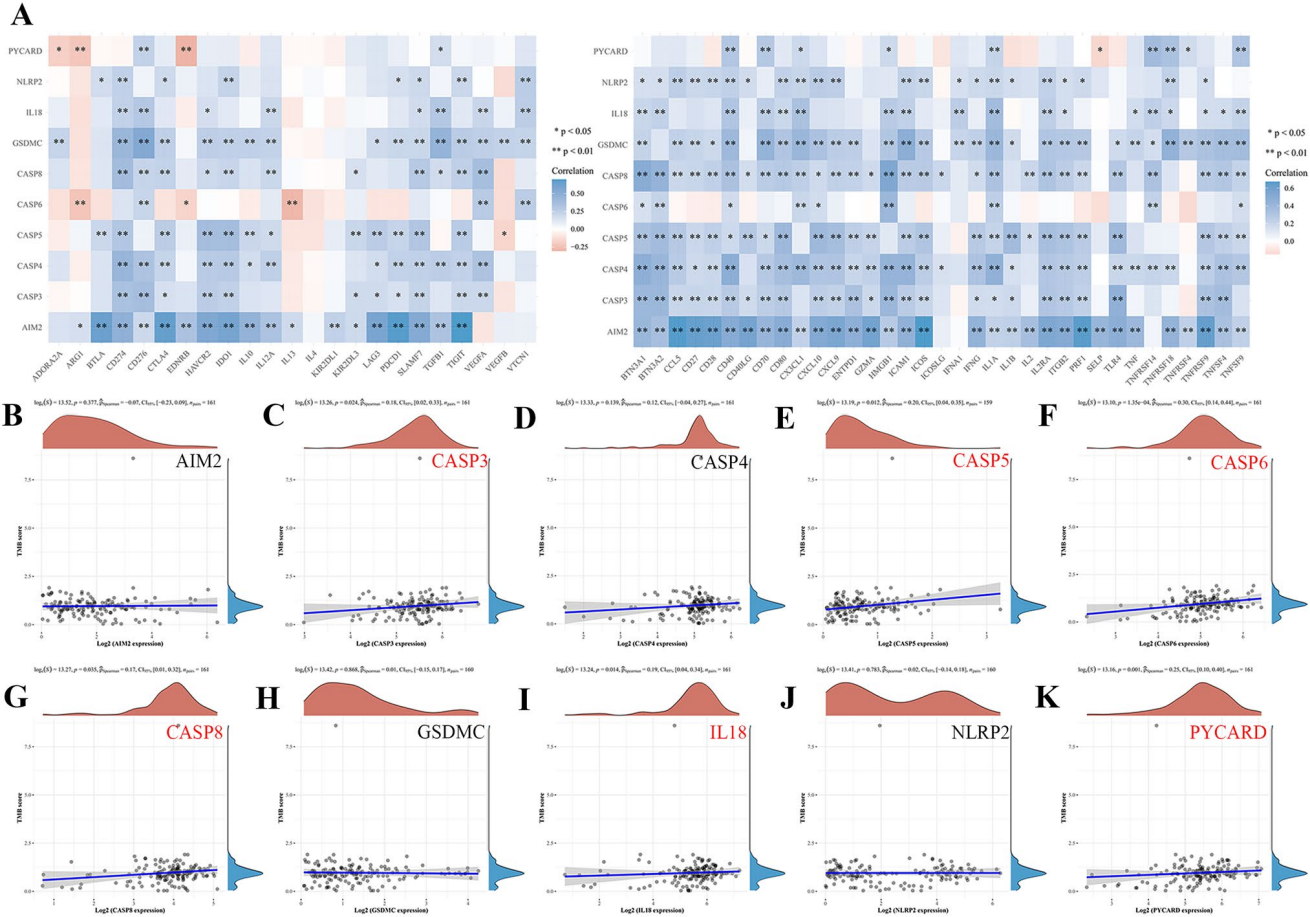
Immune checkpoints and TMB analysis of PRGs in PAAD. **A** The correlation between 10 prognostic PRGs and immune checkpoints in PAAD. **B**–**K** The correlation between 10 prognostic PRGs and TMB in PAAD. TMB, tumour mutation burden. *P < 0.05, **P < 0.01

Evidence has shown that TMB is a predictive biomarker for cancers [17]. As shown in Fig. 5A, we found that these prognostic 10 genes were positively correlated with various immune inhibitors and activators, except CASP6. We also investigated the correlation between these prognostic genes and TMB in PAAD to evaluate whether PRGs play a vital role in immunological therapy. The results showed that TMB was positively correlated with CASP3 (Fig. 5C, *P* = 0.024), CASP5 (Fig. 5E, *P* = 0.012), CASP6 (Fig. 5F, *P* = 0.000135), CASP8 (Fig. 5G, *P* = 0.035), IL18 (Fig. 5I, *P* = 0.014), and PYCARD (Fig. 5K, *P* = 0.001), but had no significant relationship with other AIM2 (Fig. 5B, *P* = 0.377), CASP4 (Fig. 5D, *P* = 0.139), GSDMC (Fig. 5H, *P* = 0.868), and NLRP2 (Fig. 5J, *P* = 0.783). In addition, we evaluated the value of immune-infiltrating cells in prognostic PRGs. We observed a positive correlation between AIM2 expression and the abundance of B cells (*P* = 9.35e−7), CD8 + T cells (*P* = 1.61e−3), CD4 + T cells (*P* = 6.95e−8, cor = 0.4), macrophages (*P* = 1.98e−5), neutrophils (*P* = 1.65e−8), and dendritic cells (*P* = 2.73e−12) (Fig. S1A). CASP3 (Fig. S1B), CASP4 (Fig. S1C), CASP5 (Fig. S1D), and CASP8 (Fig. S2A) expression was associated with immune infiltration of CD4 + T cells. Moreover, the expression of CASP6 was positively correlated with B cells (*P* = 6.94e−3), CD8 + T cells (*P* = 4.91e−4), and dendritic cells (*P* = 3.35e−2) and negatively correlated with CD4 + T cells (*P* = 3.97e−2) (Fig. S1E). GSDMC (Fig. S2B) and IL18 (Fig. S2C) expression were positively associated with CD8 + T cells, neutrophils, and dendritic cells. PYCARD was negatively correlated with CD8 + T cells (*P* = 2.14e−3) and macrophages (*P* = 4.73e−4) and positively correlated with CD4 + T cells (*P* = 1.13e−3) (Fig. S2E). However, NLRP2 expression was not significantly different between immune-infiltrated cells (Fig. S2D). These results showed that there was a significant correlation between PRGs and tumor immune infiltration.

### Identification of CeRNA (LncRNA–miRNA–mRNA) regulatory axis

**Fig. 6:**
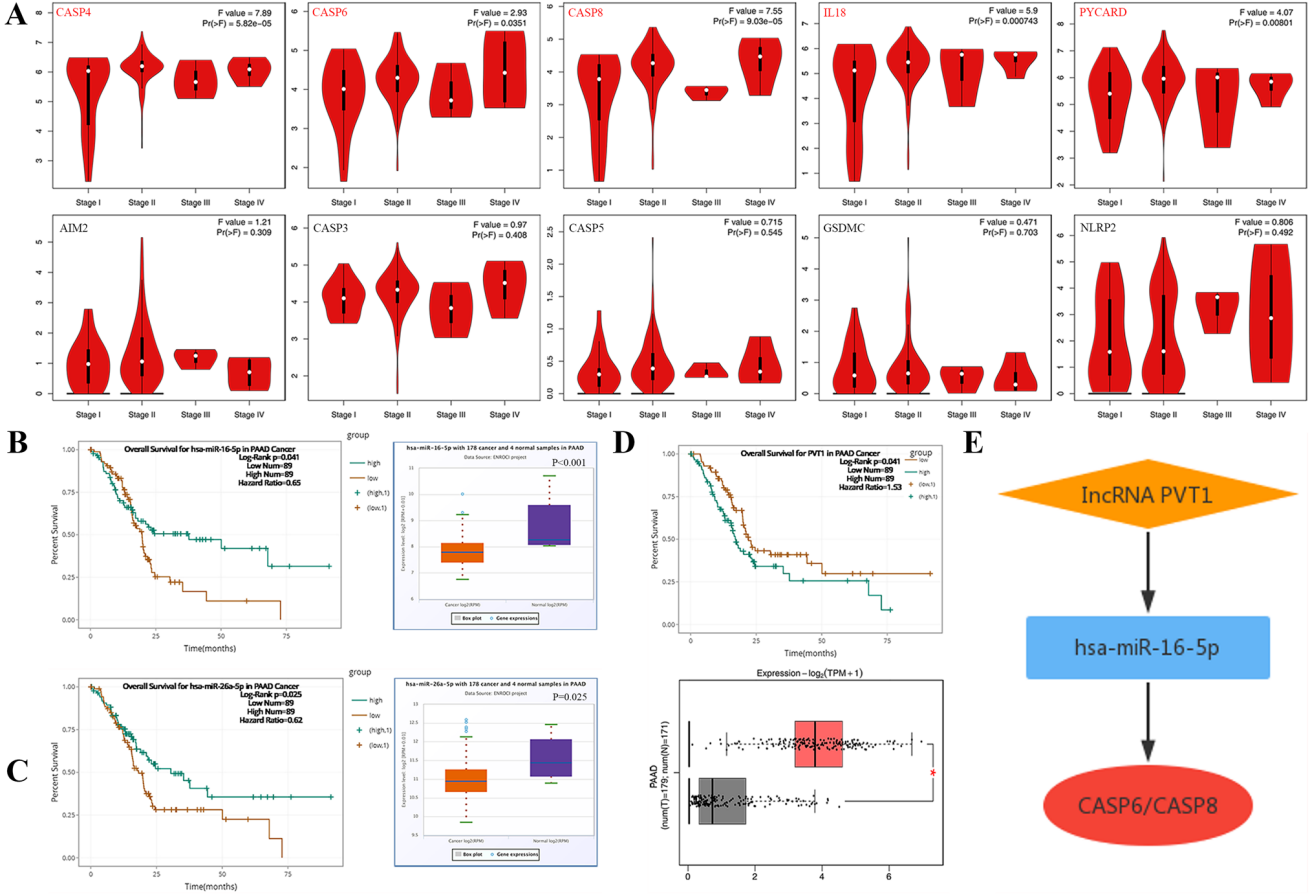
Construction of potential ceRNA network regulation axis in PAAD. **A** The association between 10 prognostic PRGs and clinical stage. **B** The overall survival and expression of hsa-miR-16-5p in PAAD. **C** The overall survival and expression of hsa-miR-26a-5p in PAAD. **D** Thr Overall survival and expression of lncRNA PVT1 in PAAD. **E** The schematic diagram of potential ceRNA network regulation axis. *P < 0.05

First, we elucidated the association between prognostic PRGs and the clinical stage (Fig. 6A). The results showed that the clinical stage was closely related to CASP4 (*P* = 5.82e−5), CASP6 (*P* = 0.0351), CASP8 (*P* = 9.03e−5), IL18 (*P* = 0.000743), and PYCARD (*P* = 0.00801), but not to AIM2 (*P* = 0.309), CASP3 (*P* = 0.408), CASP5 (*P* = 0.545), GSDMC (*P* = 0.703), and NLRP2 (*P* = 0.703). Based on the above five significant genes, we used the TarBase V.8 database to predict the upstream miRNAs, obtaining 94 mRNA-miRNA pairs (Supplementary Table 2). We further assessed the expression and prognosis of the predicted miRNAs in PAAD using starBase databases and found that only hsa-miR-16-5p (the upstream miRNA of CASP6 and CASP8) (Fig. 6B, *P* < 0.001) and hsa-miR-26a-5p (the upstream miRNA of PYCARD) (Fig. 6C, *P* = 0.025) were correlated with poor OS in PAAD patients (Fig. 6B, *P* = 0.041, Fig. 6C, *P* = 0.025). Moreover, we explored hsa-miR-16-5p and hsa-miR-26a-5p upstream lncRNAs using the miRNet database and identified 155 miRNA-lncRNA pairs (Supplementary Table 3). Finally, we analyzed the expression and prognosis of these lncRNAs in PAAD using GEPIA and starBase databases. The results showed that only the upregulation of PVT1 (upstream lncRNA of hsa-miR-16-5p) (Fig. 6D, *P* < 0.05) had a worse prognosis in patients with PAAD (*P* = 0.041). Therefore, the lncRNA PVT1/hsa-miR-16-5p/CASP6/CASP8 regulatory axis may be a critical ceRNA network in the progression of PAAD (Fig. 6E).

### Knockdown of CASP6 suppressed proliferation, migration, and invasion on PANC-1 Cell in vitro

**Fig. 7:**
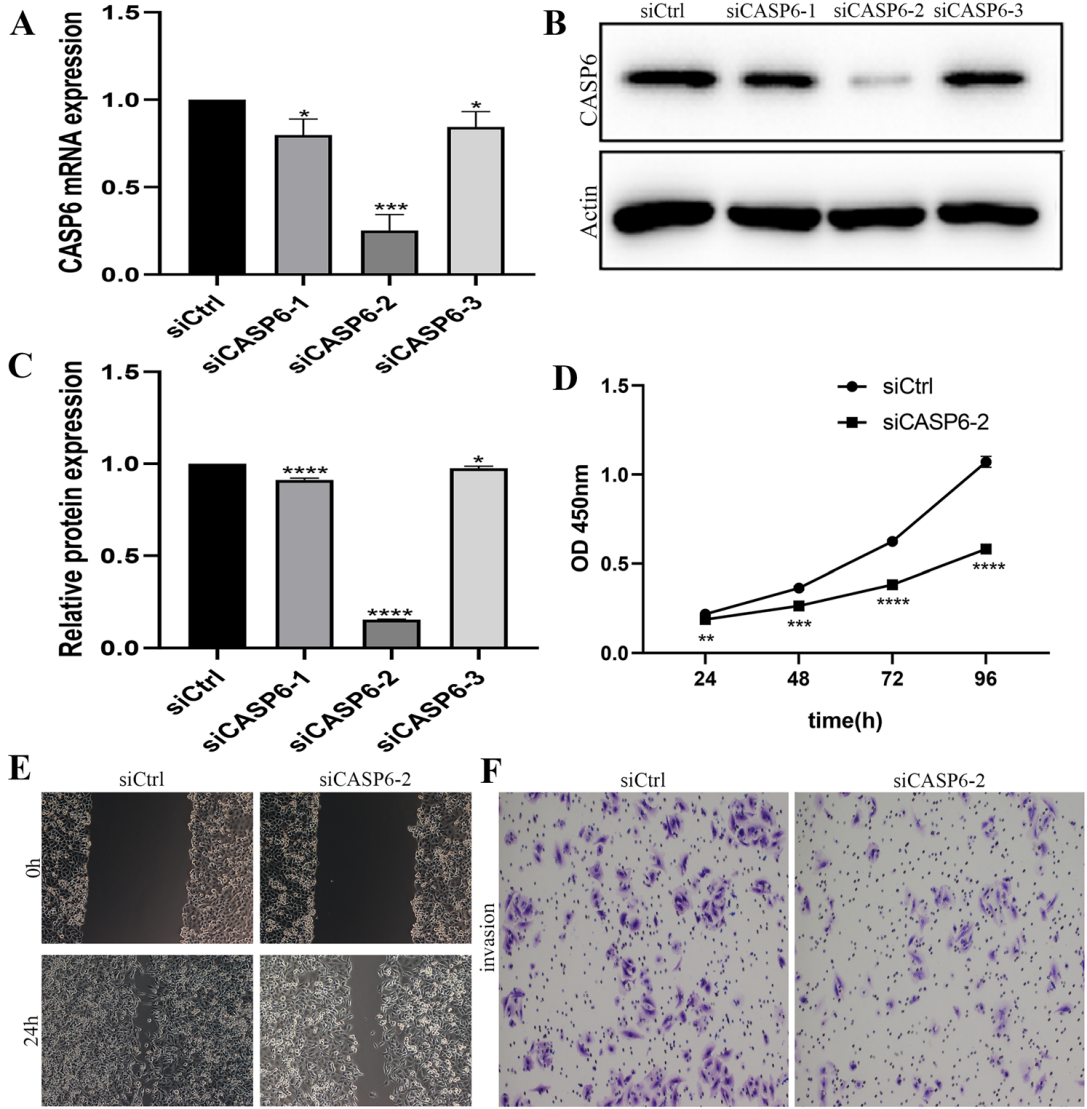
CASP6 affects PANC-1 cell proliferation, migration, and invasion. **A** Knockdown efficiency verification by qRT-PCR analysis. **B**–**C** Knockdown efficiency verification by Western blotting. **D** CCK-8 assay was performed to estimate PANC-1 cell proliferative ability upon CASP6 silencing.** E** Wounding Healing assay detected migration of PANC-1 cell upon CASP6 silencing.** F** Transwell assay detected invasion of PANC-1 cell upon CASP6 silencing. *P < 0.05, **P < 0.01, ***P < 0.001, ****P < 0.0001

The efficiency of knockdown CASP6 in PANC-1 cell was verified by qRT-PCR and Western blotting, and siCASP6-2 group with the better silence effect were used for further analyses (Fig. 7A–C). In Fig. 7D, CASP6 knockdown inhibited the activity of PANC-1 cell in the CCK-8 assay (*P* < 0.05). The wound healing assay demonstrated that the knockdown of CASP6 obviously reduced the migratory (*P* < 0.05) ability of PANC-1 cell (Fig. 7E). Moreover, the transwell assay revealed that the cell numbers of invasion was dramatically reduced in siCASP6-2 compared to control group (*P* < 0.05) (Fig. 7F).

## Discussion

Pyroptosis is a novel form of programmed and inflammatory death, with two roles in the regulation of tumorigenesis. On the one hand, pyroptosis modulates the inflammatory response and triggers malignant transformation of normal cells [18]. In contrast, pyroptosis also activates anti-neoplastic immunity, making it a potential prognostic and therapeutic target for cancers [19]. At present, a growing number of studies have focused on PRGs supported by public data and have suggested that they can serve as potential biomarkers for prognosis in patients with liver cancer [20], breast cancer [21], and skin cutaneous melanoma [22]. However, the role and potential regulation of PRGs in PAAD remain ambiguous.

In this study, we explored the expression of 33 PRGs in PAAD and normal tissues, of which 31 were differentially expressed. We then screened 10 PRGs with prognostic value, namely, AIM2, CASP3, CASP4, CASP5, CASP6, CASP8, GSDMC, IL18, NLRP2, and PYCARD, which were negatively associated with prognosis in patients with PAAD. Subsequently, based on the LassoCox regression analysis, we established a novel 4 PRG (CASP4, GSDMC, IL18 and NLRP2) signature to further evaluate the overall survival of PAAD patients. Moreover, we constructed a predictive nomogram that can provide guidance for clinical diagnosis and therapy. In addition, we investigated the PRGs that correlated with pathological stages in PAAD and developed a ceRNA network to regulate their occurrence and progression. Besides, to further confirm the tumor-promoting effect of CASP6 in PAAD, we carried out a series of experiments in vitro. The results showed that CASP6 knockdown significantly inhibited proliferation, migration and invasion in PANC-1 cells.

A nomogram has been extensively applied to determine the probability of a clinical event by integrating different variables [23]. Previous studies have constructed several prognostic signatures from different perspectives in PAAD. For example, Ding et al. identified that three apoptosis-related genes are closely related to the sensitivity of PAAD to chemotherapy [24]. A recent retrospective study indicated that high level of CA199, neutrophil–lymphocyte ratio, lymph node metastasis, and distant metastasis were independent prognostic factors in patients with PAAD [25]. A further study constructed an autophagy-related prognostic model and considered it a therapeutic target for PAAD [26]. Our study first constructed a novel 4 PRG signature (CASP4, GSDMC, IL18, and NLRP2) in PAAD, which provides a new choice and direction for the prognosis of patients. CASP4 is important in typical and atypical inflammasome-induced pyroptosis [27]. After activation, CASP4 directly cleaves GSDMD and releases IL-18, which promotes CASP1 activation, leading to pyroptosis [28]. CASP4 can affect the progression of ccRCC and PAAD and can be used as a potential prognostic biomarker [29, 30]. Pyroptosis is a form of GSDM-mediated programmed necrosis and activated caspase-8 induces pyroptosis by cleaving GSDMC [31]. TNFα-activated caspase-8 converts apoptosis to pyroptosis by cleaving PD-L1-induced GSDMC [32]. A growing number of studies have reported that GSDMC expression is increased in a variety of cancers, including CRC [33], LUAD [34], and KIRC [35], and knockdown of GSDMC can inhibit the biological behavior of KIRC and CRC. IL-18, a pro-inflammatory cytokine, influences the tumor microenvironment, tumor progression, metastatic dissemination, and sensitive resistance [36, 37]. NLRP2 forms an inflammatory complex with ASC and caspase-1 and regulates NF-κB activity [38]. It has been demonstrated that NLRP2 is closely associated with the prognosis of AML [39] and HNSC [40]. These results further confirm the potential prognostic value of the PRGs signature in PAAD. However, further experiments are required to verify the exact mechanism by which they participate in pyroptosis in PAAD.

The tumor microenvironment (TME) plays a central role in cancer immunosuppression, with a physiological state highly relevant to tumorigenesis and progression [41, 42]. The Gasdermin (GSDM) family member genes NLRP3, NLRC4, NLRP1, AIM2, IL-1β, and IL-18 are linked to TIME, and the immunosuppressive microenvironment can be overcome by targeted pyroptosis therapy in cancers [43]. We explored the relationship between ten valuable prognostic genes in PRGs and immune cell infiltration in PAAD. It was found that most of the PRGs prognostic genes were positively correlated with the infiltration of many types of immune cells, which indicated that the high expression of PRGs was related to high immune activity in the tumor microenvironment of PAAD. Pyroptosis may promote the accumulation of immune cells and regulate the composition of the tumor immune microenvironment, leading to the occurrence of PAAD. Moreover, we investigated the relationship between prognostic genes, immune checkpoints, and TMB. We demonstrated that the prognostic genes were positively correlated with most of the immune checkpoints except CASP6. And TMB was positively correlated with CASP3, CASP5, CASP6, CASP8, IL18 and PYCARD. Increasing evidence has shown that patients with high TMB may benefit from immunotherapy, which indicates that CASP3, CASP5, CASP6, CASP8, IL18, and PYCARD genes have the potential to be used as biomarkers for predicting immunotherapy in patients with PAAD and may provide a new perspective for the development of PAAD treatment.

To elucidate the mechanism of PRGs in the progression of PAAD, we constructed an mRNA-miRNA-lncRNA network based on the underlying ceRNA hypothesis using online databases. CASP4, CASP6, CASP8, IL18, and PYCARD were associated with clinical stage, which means that IL18 and PYCARD may be involved in tumor progression. We identified the PVT1/hsa-miR-16-5p/CASP6/CASP8 regulatory axis. PVT1 sponges miR-20a-5p to act on NLRP3-mediated pyroptosis [44]. Knockdown of lncRNA PVT1 can reduce pyroptosis in cardiomyocytes by targeting GSDMD [45]. Furthermore, PVT1 contributes to tumor development by targeting miR-519d-3p/HIF-1A in PAAD [46]. Importantly, our study is the first to demonstrate that PVT1 directly regulates the expression of CASP6 and CASP8 through hsa-miR-16-5p, thereby affecting pyroptosis.

In addition, an important finding of this study is that CASP6, a key regulator of innate immune inflammation activation and host defense [47]. Increasing evidence has revealed that CASP6 is involved in carcinogenesis and tumor progression, such as glioma [48] and colorectal cancer [49]. However, the status of CASP6 as a PRG in PAAD has been rarely studied, therefore, the role of CASP6 in PAAD was unclear. Our results showed that CASP6 is one of the biomarkers of PRGs in the prognosis of PAAD. Patients with higher CASP6 expression had shorter survival in the OS analysis, and CASP6 was closely associated with clinical stage. CASP6 may be a potential prognostic marker and therapeutic target in the immune microenvironment of PAAD. In vitro functional assays are consistent with the results of bioinformatics methods, further confirming that CASP6 may be a potential target for the treatment of PAAD.

Although the model has shown potential clinical value in PAAD, there are several limitations to this study. Firstly, the tumor tissue samples in TCGA database were comparatively limited, and more samples are needed for further validation. Secondly, an external validation cohort is required to verify the clinical applicability of the model. Thirdly, additional in vivo and in vitro experiments are needed to provide insight into the molecular mechanisms in the future.

## Conclusion

The PRGs were associated with prognosis, TMB, immune checkpoints, and immune infiltration, and CASP6 could be a potential biomarker, promoting the occurrence and progression in PAAD. In vitro, CASP6 was validated as an oncogene in PAAD, and CASP6 inhibition prevented PANC-1 cells proliferation, migration and invasion. In addition, we first identified a critical ceRNA regulatory axis lncRNA PVT1/hsa-miR-16-5p/CASP6/CASP8 in PAAD progression. This study preliminarily revealed the potential biomarkers and molecular mechanisms of PAAD progression, which is beneficial for the discovery of therapeutic targets.

## Supplementary Information

Below is the link to the electronic supplementary material.

Fig. S1 The association between 5 prognostic PRGs and immune infiltration in PAAD A AIM2 B CASP3 C CASP4 D CASP5 E CASP6. (TIF 21367 kb)

Fig. S2 The association between 5 prognostic PRGs and immune infiltration in PAAD A CASP8 B GSDMC C IL18 D NLRP2 E PYCARD. (TIF 21390 kb)

Supplementary file3 (XLSX 9 kb)

Supplementary file4 (XLSX 11 kb)

Supplementary file5 (XLSX 11 kb)

## Data Availability

The datasets presented in this study can be found in online repositories. The names of the repository/repositories and accession number(s) can be found in the article/Supplementary Material.
