# Supplementary figures and images for: Dissection of pyroptosis-related prognostic signature and CASP6-mediated regulation in pancreatic adenocarcinoma: new sights to clinical decision-making
Source: Apoptosis. 2023 Mar 7;28(5-6):769–82. doi: 10.1007/s10495-023-01823-7 (PMC10232623; doi:10.1007/s10495-023-01823-7)

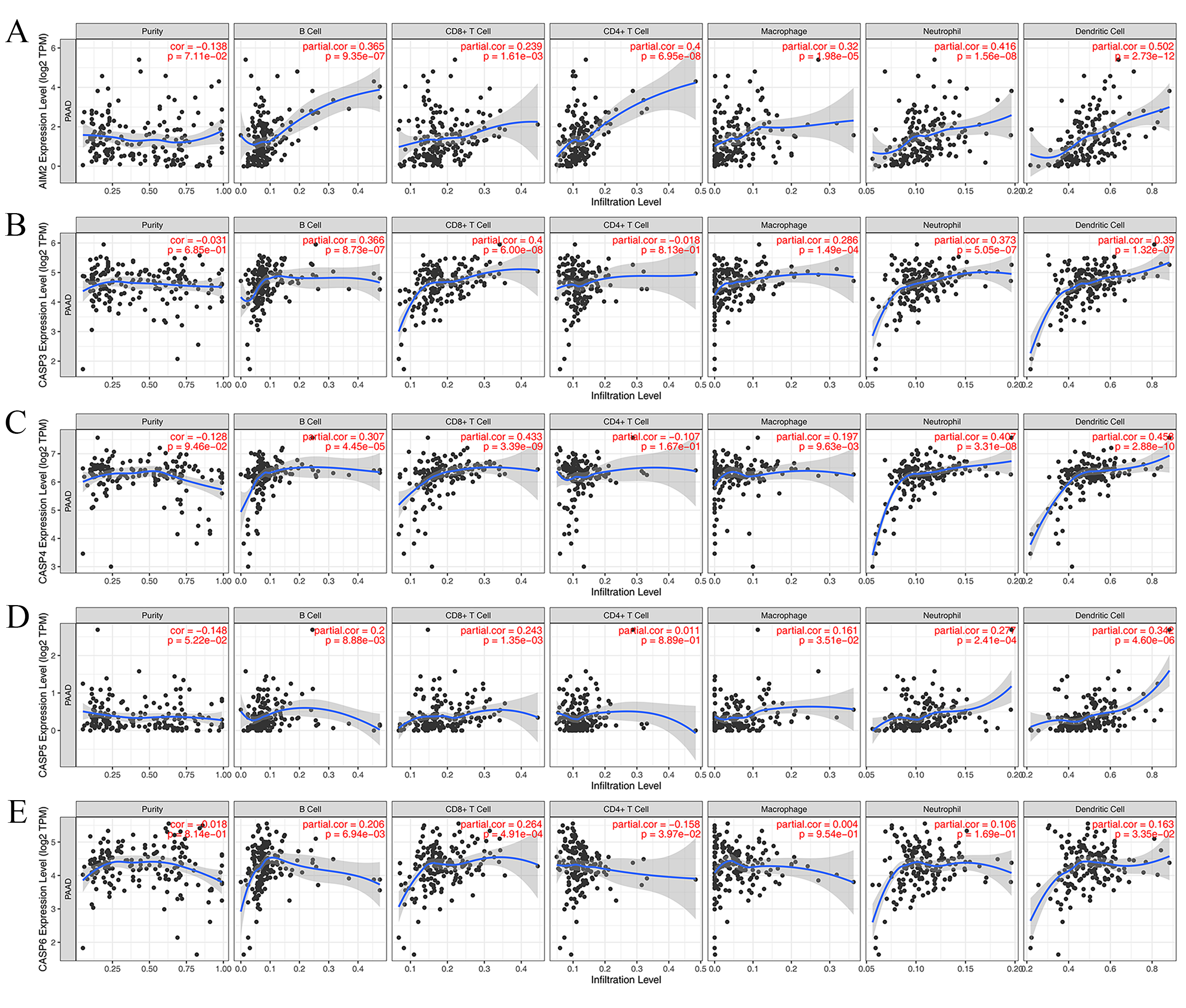

Supplement: Supplementary file 1 — Fig. S1 The association between 5 prognostic PRGs and immune infiltration in PAAD A AIM2 B CASP3 C CASP4 D CASP5 E CASP6. (TIF 21367 kb) [file 10495_2023_1823_MOESM1_ESM.tif]

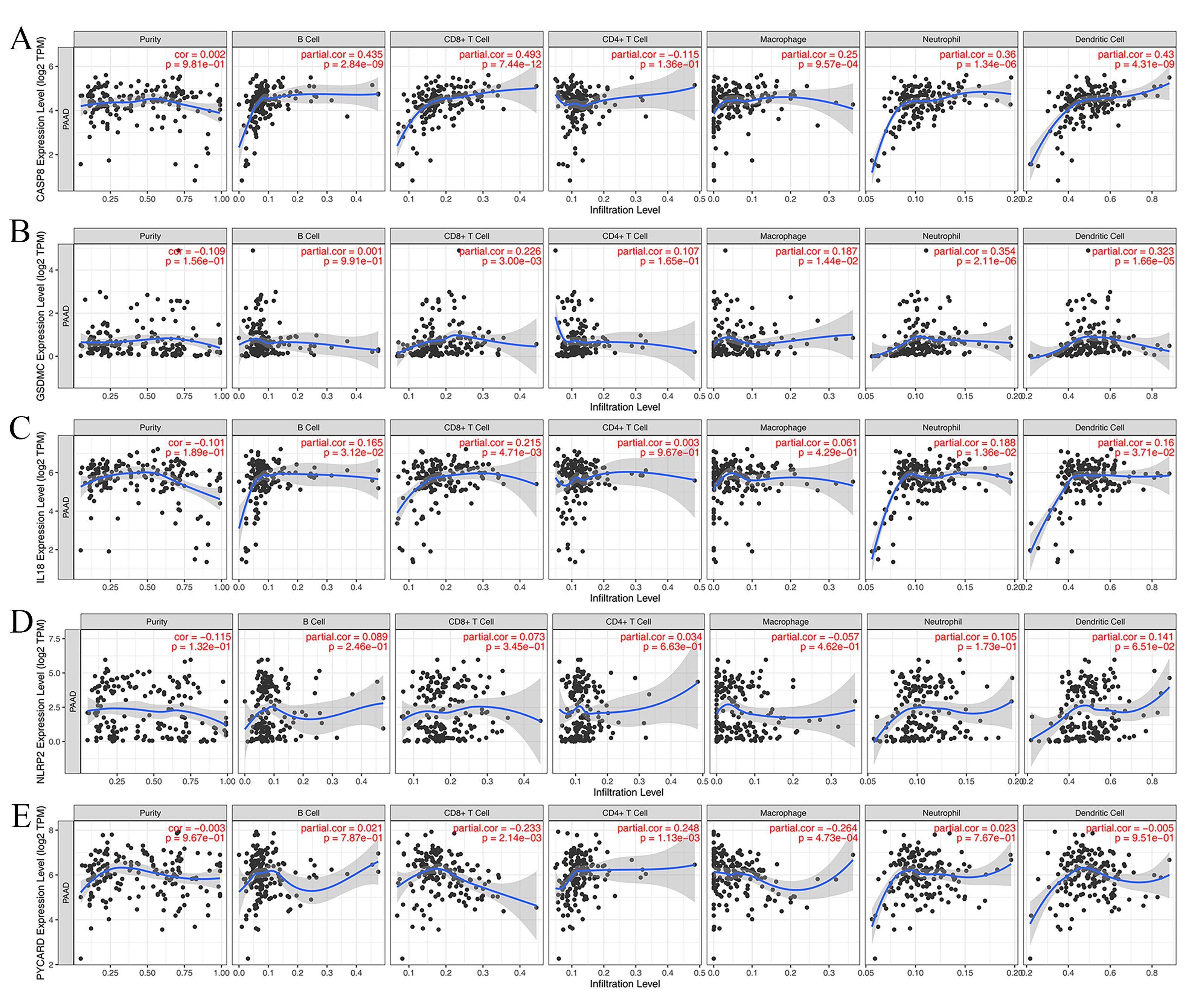

Supplement: Supplementary file 2 — Fig. S2 The association between 5 prognostic PRGs and immune infiltration in PAAD A CASP8 B GSDMC C IL18 D NLRP2 E PYCARD. (TIF 21390 kb) [file 10495_2023_1823_MOESM2_ESM.tif]
